# Usefulness of Artificial Pneumothorax during Totally Endoscopic Off-Pump Left Atrial Appendage Closure and Surgical Ablation

**DOI:** 10.5761/atcs.oa.24-00156

**Published:** 2025-01-28

**Authors:** Shunsuke Sato, Takashi Azami, Jun Fujisue, Kyozo Inoue, Kenji Okada

**Affiliations:** 1 Department of Cardiovascular Surgery, Yodogawa Christian Hospital, Osaka, Osaka, Japan; 2 Department of Cardiovascular Surgery, Kobe University, Kobe, Hyogo, Japan

**Keywords:** artificial pneumothorax, atrial fibrillation, minimally invasive cardiac surgery, left atrial appendage closure, maze

## Abstract

**Purpose:** In totally endoscopic off-pump left atrial appendage (LAA) closure and surgical ablation, securing the operative field is sometimes difficult in some patients because of a narrow working space caused by an elevated diaphragm or ventricles. In this study, we aimed to investigate the effectiveness of a method that facilitates securing the operative field using an artificial pneumothorax.

**Methods:** We analyzed 71 consecutive patients who underwent totally endoscopic off-pump LAA closure and bilateral pulmonary vein isolation. The factors contributing to the reduction in operative time were examined. The patients were divided into the following 2 groups according to whether or not an artificial pneumothorax was used: Group C comprised 24 patients without an artificial pneumothorax and Group A comprised 47 patients with an artificial pneumothorax.

**Results:** There were no hospital deaths or major complications. The operative time was significantly shorter in Group A (108 ± 26 minutes) than in Group C (198 ± 77 minutes) (p <0.0001).

**Conclusions:** In totally endoscopic off-pump LAA closure and surgical ablation, an artificial pneumothorax may be useful in reducing the operative time.

## Introduction

Totally endoscopic off-pump left atrial appendage closure and surgical ablation (TEOLA)^1–3)^ is a minimally invasive and innovative procedure. Securing the operative field is sometimes difficult in some patients because of a narrow working space caused by an elevated diaphragm or large ventricles. Although we sometimes face technically difficult cases in this surgery, there have been few reports that provide technical support. In the present study, we aimed to investigate the effectiveness of a method that facilitates securing the operative field using an artificial pneumothorax along with a check valve on all ports used.

There have been reports of the use of artificial pneumothorax in respiratory surgery^4)^ and esophageal surgery,^5)^ but as far as searching PubMed abstract, there are no reports on the use of artificial pneumothorax in cardiac surgery.

## Materials and Methods

The use of the device and the study design were approved by our institution’s ethics committee, and all patients provided written informed consent before the procedure.

We analyzed 71 consecutive patients who underwent port wound only, off-pump, right and left pulmonary vein isolation, and left atrial appendage (LAA) closure between November 2018 and November 2023 at our institution. The factors contributing to the reduction in operative time were examined. The patients were divided into the following 2 groups according to the use of artificial pneumothorax: Group C comprised 24 patients without an artificial pneumothorax and Group A included 47 patients with an artificial pneumothorax (**Table 1**). The use of artificial pneumothorax depended on the time of year; an artificial pneumothorax was not used in all cases until March 2022, and an artificial pneumothorax was used in all cases after April 2022.

**Table 1:** Patients’ baseline characteristics

Characteristic	All	Group C	Group A	p-Value
Patients	71	24	47	
Female, n (%)	19 (27)	8 (33)	11 (23)	0.3713
Age, years	67 ± 10	65 ± 9	68 ± 10	0.2823
Previous cardiac/pulmonary surgery or thoracic radiotherapy	4 (6)	2 (8)	2 (2)	0.4808
History of catheter ablation	8 (11)	2 (8)	6 (13)	0.5827
Type of atrial fibrillation, n (%)				0.2033
Paroxysmal	20 (28)	4 (17)	16 (34)	
Persistent	12 (17)	6 (25)	6 (13)	
Longstanding persistent	39 (55)	14 (58)	25 (53)	
Echocardiography				
LVDd, mm	47 ± 6	46 ± 7	47 ± 6	0.4741
LVEF	57 ± 10	59 ± 11	57 ± 10	0.5209
LADs, mm	45 ± 8	44 ± 8	45 ± 8	0.6074

Continuous data are presented as mean ± standard deviation and categorical data as numbers (%).

LVDd: left ventricular end-diastolic diameter; LVEF: left ventricular ejection fraction; LADs: left atrial dimension at the end-systolic phase

### Procedure

**Fig. 1:**
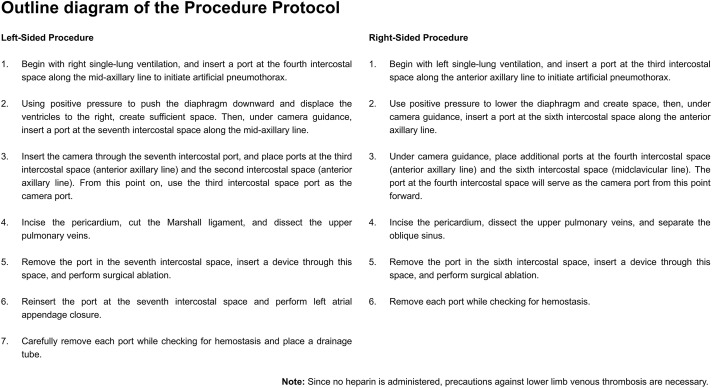
Outline diagram of the procedure protocol.

The procedure was performed in the supine position with ports from both lateral chests while using one-lung ventilation (OLV; **Fig. 1**). A total of 4 ports were used, with diameters of 12, 11, 10, and 5 mm. The most caudal port on both the left and right sides was 12 mm in diameter; thus, this was used as the main port. When an artificial pneumothorax was used, ports with check valves were used, except for the camera port. The check valve on the camera port was not necessary because the camera was always inserted and not taken in or out. The positive pressure for the artificial pneumothorax was 5–8 mmHg (**Supplementary Video**).

Bilateral pulmonary vein isolation was performed in all patients, and en bloc left pulmonary vein and appendage isolation (EBLI)^1)^ was added in cases where it was technically safe.

LAA management was performed by closure with a clip device (AtriClip PRO2 device; Atricure Inc. Mason, OH, USA) or by resection with a stapler device (ECHELON FLEX Powered ENDOPATH Stapler 60; Ethicon, Cincinnati, OH, USA). Both devices and the sizer for measurement passed through a 12-mm port; thus, the positive pressure of the artificial pneumothorax could be easily maintained during left heart processing.

The AtriCure RF ablation system (Atricure Inc.) was used for surgical ablation, and the AtriCure Dissector (Atricure Inc.) was used to guide the devices. Given that these devices do not pass through a port, the port was removed, the device was inserted directly through the wound, and positive pressure was maintained by manually compressing the device insertion site.

In LAA management, resection with a stapler device was chosen for cases with an LAA base of >50 mm, with a thrombus located at the tip of the LAA, or a desirable LAA pathology.

In cases with a high risk of bleeding from the LAA transection and in cases with difficult LAA processing operations, closure with a clip device was selected.

However, because the clip device only became available in Japan in 2022, the stapler device was used in all cases operated prior to that year. The clip device is only available in sizes up to 50 mm; thus, it cannot be used for cases with a large LAA. In the case of the stapler device, complete resection was achieved with multiple firings, even in cases where the base of the left auricle was too long to be resected in 1 session. When the stapler device was used for resection, a ligation closure with a loop device was added when the LAA remained caudally due to the approach angle.

In cases with a thrombus in the LAA tip, the clip device was not used because its application required touching the LAA tip, and there was a risk of scattering the thrombus. Thus, resection with a stapler device was used because the device can be applied without touching the LAA tip.

In cases where the LAA thrombus was large and extended near the LAA base, thrombus removal and LAA management were performed using a cardiopulmonary bypass; these cases were excluded from this study.

This procedure is consistently performed by a single surgeon.

### Statistical analysis

JMP12.0.1 (SAS Institute Inc., Cary, NC, USA) was used for all statistical analyses, and p-values <0.05 were considered significant. Pearson’s chi-square test was used to examine the nominal measures. The variability of the operative time was examined by performing an analysis of variance. The stepwise methods were used for multivariate analysis.

## Results

There were no hospital deaths in the entire study series. There were no major complications, such as cerebral infarction, cerebral hemorrhage, conversion to an open chest, or reopened hemorrhage. Compared with Group C, Group A had a higher percentage of left main port position in the 7th or 8th intercostal space, clip device use, an EBLI, and a shorter operative time (**Table 2**). The analysis of variance showed a predominance of factors in Group A for operative time, and Group A had a stable operative time (**Table 2**).

**Table 2:** Results

Characteristic	All	Group C	Group A	p-Value	F
Left main port location, n (%)				<0.0001	
Sixth intercostal space	22 (31)	19 (79)	3 (6)		
Seventh or eighth intercostal space	49 (69)	5 (21)	44 (94)		
LAA management, n (%)				0.0276	
Closure with clip device	17 (24)	2 (8)	15 (32)		
Resection with stapler device	54 (76)	22 (92)	32 (68)		
Additional loop ligation	2 (4)	2 (9)	0	0.0822	
En Bloc Left PV and LAA Isolation, n (%)	37 (52)	6 (25)	31 (66)	0.0011	
Operation time, minutes	138 ± 65	198 ± 77	108 ± 26	<0.0001	F (1, 26) = 31, p <0.0001*
Blood transfusion, n (%)	1 (1)	1 (4)	0	0.1587	

Continuous data are presented as mean ± standard deviation and categorical data as numbers (%).

*F (1, 26) = 31, p <0.0001.

The F-value indicates the difference in variance between groups. The larger the F-value, the more the variance in surgical time differs depending on the presence or absence of artificial pneumothorax. Since p <0.0001, this indicates that there is a significant difference in the variance of surgical time depending on the presence or absence of artificial pneumothorax. Specifically, it can be interpreted that artificial pneumothorax is likely to reduce the variability in surgical time.

LAA: left atrial appendage; PV: pulmonary vein

Univariate analysis demonstrated that the factors contributing to the shorter operative time were artificial pneumothorax, left main port in the 7th or 8th intercostal space, use of clip device, and EBLI (**Table 3**). The multivariate analysis of these factors showed that the predominant factors were the presence of artificial pneumothorax and the left main port in the 7th or 8th intercostal space (**Table 4**).

**Table 3:** Univariate analysis for surgery time

Characteristic	n	Operation time, minute	p-Value
Sex			
Female	19	134 ± 15	
Male	52	140 ± 9	0.7476
Artificial pneumothorax			
Not used	24	198 ± 10	
Used	47	108 ± 7	<0.0001
Left main port location			
Sixth intercostal space	22	199 ± 11	
Seventh or eighth intercostal space	49	111 ± 7	<0.0001
LAA management			
Closure using clip	17	106 ± 16	
Resection using stapler	54	148 ± 9	0.0175
Resection only	52	146 ± 10	
Additional loop ligation	2	225 ± 45	0.1192
En Bloc Left PV and LAA Isolation			
Not performed	34	157 ± 11	
Performed	37	121 ± 10	0.0210

Continuous data are presented as mean ± standard deviation and categorical data as numbers (%). LAA: left atrial appendage: PV: pulmonary vein

**Table 4:** Multivariate analysis for surgery time

Characteristic	p	Sum of squares	R-dimensional	Cp	AIC
Artificial pneumothorax	<0.01	129586	0.44	5.6	759
Left main port position in 7th/8th ICS	0.02	12380	0.48	2.2	755
LAA management (closure or resection)	0.37	1814	0.49	3.4	757
En bloc left PV and LAA isolation	0.51	1009	0.49	5.0	758

AIC: Akaike’s information criterion; CP: process capability index; ICS: intercostal space; LAA: left atrial appendage; PV: pulmonary vein

Throughout the LAA closure procedure, we continuously monitored with transesophageal echocardiography, and no air embolisms into the left atrium were observed.

At 1-month post-op, sinus rhythm restoration rates were 91% for paroxysmal atrial fibrillation (AF), 78% for chronic AF less than a year, and 98% for chronic AF between 1 and 5 years. However, patients with chronic AF over 5 years had poor restoration rates.

## Discussion

In the present study, we devised a method for TEOLA in which all ports used are equipped with a check valve and an artificial pneumothorax is used to secure the operative field. There have been reports of the use of artificial pneumothorax in respiratory surgery^4)^ and esophageal surgery,^5)^ but as far as searching PubMed abstract, there are no reports on the use of artificial pneumothorax in cardiac surgery.

All procedures were performed in the supine position, with the bed tilted to elevate the head. For procedures on the left side, the patient was tilted as far as possible to the right, and similarly to the left for right-sided procedures.

We encountered no major issues with PaCO2. According to the anesthesiologist, artificial pneumothorax causes less PaCO2 elevation compared to abdominal insufflation. However, after performing left-sided surgery with right-lung OLV, we sometimes observed a drop in SpO2 immediately after switching to left-lung OLV for right-sided surgery. In such cases, we would temporarily pause the procedure and resume bilateral ventilation until SpO2 recovered, leading to stabilization over several repetitions. This issue likely arises because ventilation-perfusion (V/Q) matching worsens when relying on the previously collapsed lung. As time progresses with contralateral OLV, V/Q matching improves, and ventilation stabilizes. Since the patient is supine, V/Q matching may be less favorable than in lateral decubitus positioning.

A known risk of artificial pneumothorax is hypotension. With pneumothorax pressures up to 8 mmHg, continuous vasopressor infusion was sometimes needed, though there was no significant hemodynamic impact. However, blood pressure tended to drop when the pressure was raised to 10 mmHg.

**Fig. 2:**
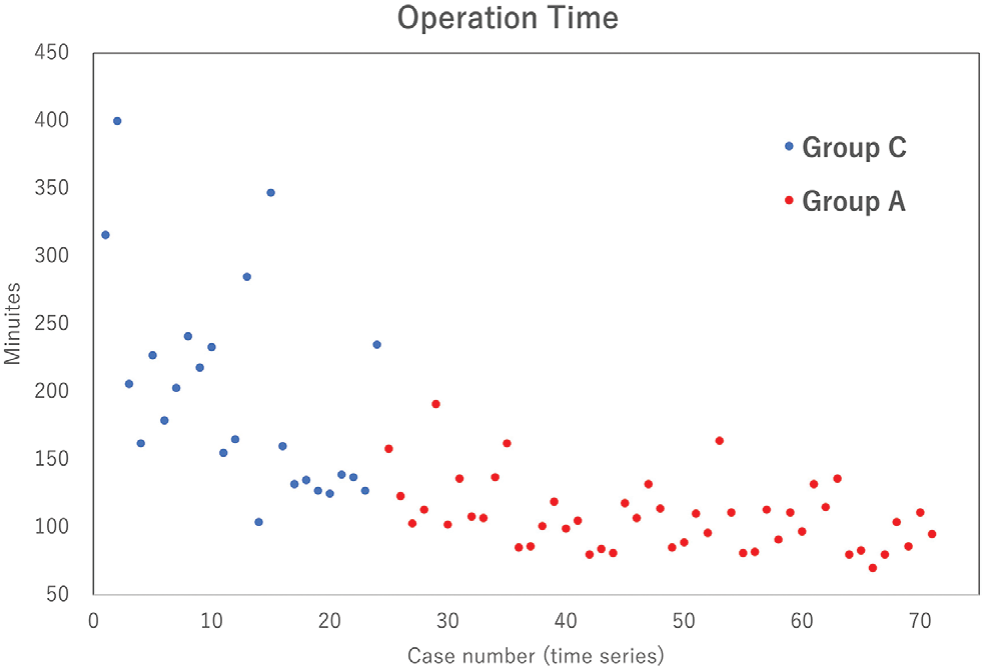
Distribution of operative time. Group C is a conventional method group that did not use artificial pneumothorax, and Group A used an artificial pneumothorax.

In our study, the variability of the operative time decreased and became stable and shorter in the artificial pneumothorax group (**Tables 2** and **3**, **Fig. 2**). The surgeon’s impression was that, before the introduction of the artificial pneumothorax, obtaining a sufficient field of vision was difficult, especially in patients with an elevated diaphragm and large left ventricle, and the operation took a long time.

The artificial pneumothorax shortened the time required for the lung to collapse sufficiently after OLV until the visual field could be secured, and the hemostatic effect of positive pressure may have also contributed to the shortening of the operative time.

**Fig. 3:**
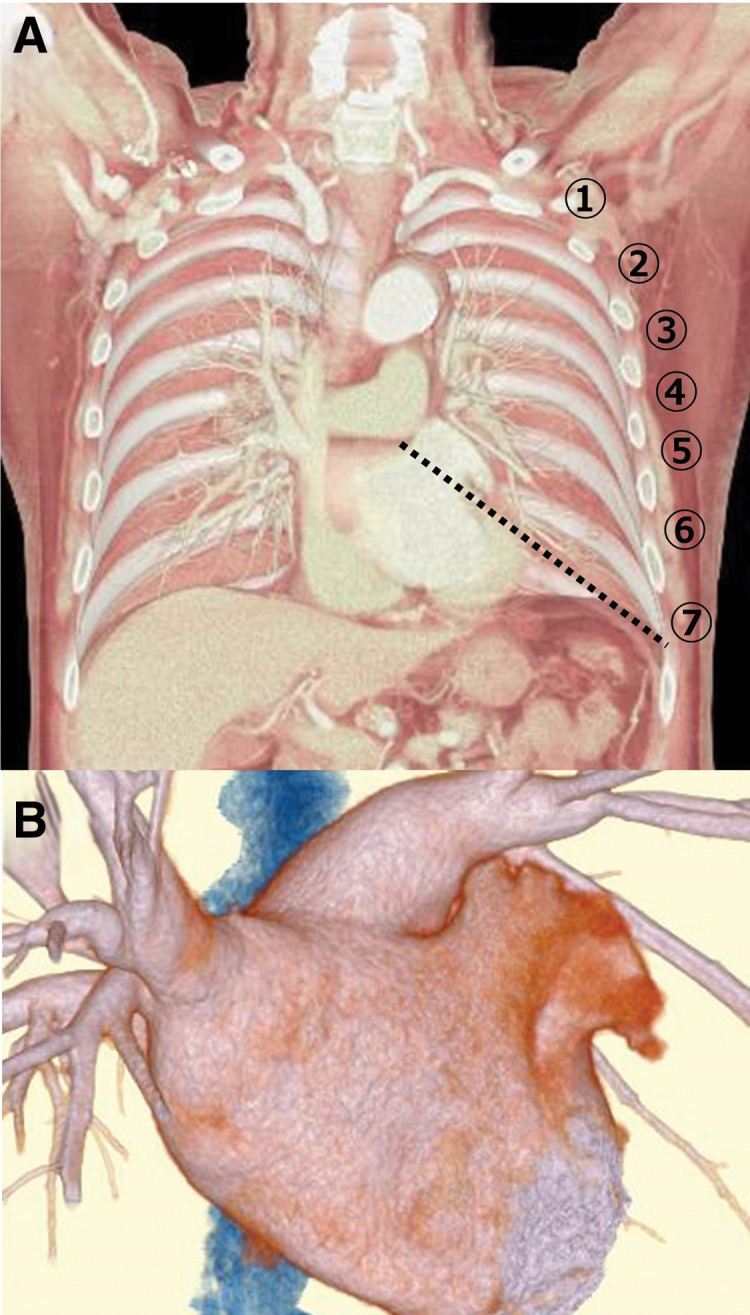
Simulation of left atrial appendage management device insertion port location. (**A**) 3D-constructed CT. The dotted line is an extension of the line at the base of the left atrial appendage. 〇Numbers represent intercostals–Example: ① is the first intercostal space. (**B**) Same case as in (**A**). Left atrium constructed in 3D using contrast-enhanced CT. CT: computed tomography

If the left main port location was caudal to the 7th or 8th intercostal space, the operative time was shorter as compared to that in the sixth intercostal space. In many cases, an extension of the straight line at the base of the left auricle on preoperative computed tomography (CT) often results in a seventh intercostal axillary midline (**Fig. 3**). However, without artificial pneumothorax, if the left thoracic cavity is opened on OLV, the diaphragm may interfere with port placement from the 7th intercostal axillary midline (**Fig. 3**); therefore, the 6th intercostal axillary midline is often chosen for the most caudal port on the left side. When a stapler device is inserted through the midline of the 6th intercostal axilla and LAA resection is performed, the LAA may be left caudally, and ligature closure with a loop may be necessary. Although there are reports on the good results with stapler and loop ligation and closure,^6)^ the closure of the residual LAA by a loop is difficult to get used to. In many cases, the device can be inserted from the caudal side of the 7th intercostal space without bending the device, and the residual LAA tail edge is eliminated; therefore, an additional procedure is unnecessary, which may have led to a reduction in operative time.

We did encounter adhesions between the lung and pleura. While some cases required adhesion dissection, no lung injuries required repair. For cases where lung adhesions were suspected, we assessed lung and pleural sliding with transthoracic ultrasound and inserted the initial port in an area deemed free of adhesions.

For LAA closure, it is easier to manipulate the device when inserted from a more caudal port. To facilitate this, we first placed a port near the axilla, used an artificial pneumothorax to lower the diaphragm, and then, under camera guidance, inserted the caudal port.

In LAA management, resection with a stapler device was chosen for cases with an LAA base of >50 mm, a thrombus present at the tip of the LAA, or a desirable LAA pathology.

In cases with a high risk of bleeding from the LAA transection and in cases with difficult LAA processing operations, closure with a clip device was selected.

However, because the clip device only became available in Japan in 2022, the stapler device was used in all cases operated prior to that year. The clip device is only available in sizes up to 50 mm; thus, it cannot be used for cases with a large LAA. In the case of the stapler device, complete resection was achieved with multiple firings, even in cases where the base of the left auricle was too long to be resected in 1 session. When the stapler device was used for resection, a ligation closure with a loop device was added when the LAA remained caudally due to the approach angle.

In the case of a thrombus in the LAA tip, the clip device was not used because its application requires touching the LAA tip when fitting, and resection with a stapler device was used because the device can be applied without touching the LAA tip.

In cases where the LAA thrombus was large and extended near the LAA base, thrombus removal and LAA management were performed using a cardiopulmonary bypass; these cases were excluded from this study.

There appears to be no difference in the long-term outcome of embolism prophylaxis between cases with a resected or closed LAA.^7)^ There was also no significant difference in the operative time, although men had thicker intercostal muscles, and device manipulation required a little more force (**Table 3**).

## Limitation

Given that the surgical timing was different between Groups C and A, the results were affected by the learning curve. However, after the introduction of artificial pneumothorax, there was a dramatic reduction in the operative time. Since AtriClipPRO2 only became available in Japan in 2022, there was a difference in the percentage of clip device use between Groups C and A. Moreover, this report is based on a retrospective analysis and is not a randomized controlled study. The case volume and follow-up length were insufficient to allow us to draw firm conclusions. Additionally, all patients were operated on by a single surgeon proficient in endoscopic surgery; hence, our clinical outcomes are too limited to allow generalizations about the safety and efficacy of our method.

## Conclusion

In conclusion, in TEOLA, the use of an artificial pneumothorax may be useful in reducing the operative time.

## Declarations

### Ethics approval and consent to participate

The institutional review board of Yodogawa Christian Hospital approved this study (2023-037); all study participants provided written informed consent for participation.

### Consent for publication

All study participants provided written informed consent for participation.

### Funding

None.

### Data availability

The data that support the findings of this study are available from the corresponding author, upon reasonable request.

### Author contributions

Azami, Fujisue, and Inoue contributed to data collection. Okada designed the study protocol. All authors read and approved the final manuscript. All authors have consented to be accountable for all aspects of the research.

### Disclosure statement

Sato received honoraria of lecture fees from Century Medical Inc. Azami, Fujisue, Inoue and Okada declare that there is no conflict of interest.

## Supplementary Material

Supplementary Video Artificial pneumothorax creates space in the left and right thoracic cavity
